# Usefulness of the Vitreous Fluid Analysis in the Translational Research of Diabetic Retinopathy

**DOI:** 10.1155/2012/872978

**Published:** 2012-09-17

**Authors:** Olga Simó-Servat, Cristina Hernández, Rafael Simó

**Affiliations:** ^1^Department of Endocrinology, Hospital Vall d'Hebron, Barcelona, Spain; ^2^Diabetes and Metabolism Research Unit, Vall d'Hebron Institute of Research (VHIR), Autonomous University of Barcelona, Pg Vall d'Hebron 119-129, 08035 Barcelona, Spain; ^3^CIBER de Diabetes y Enfermedades Metabólicas Asociadas, (CIBERDEM), Instituto de Salud Carlos III, 08035 Barcelona, Spain

## Abstract

Diabetic retinopathy (DR) is the major cause of acquired blindness in working-age adults. Current treatments for DR (laser photocoagulation, intravitreal corticosteroids, intravitreal antivascular endothelial growth factor (VEGF) agents, and vitreo-retinal surgery) are applicable only at advanced stages of the disease and are associated with significant adverse effects. Therefore, new pharmacological treatments for the early stages of the disease are needed. Vitreous fluid obtained from diabetic patients undergoing vitreoretinal surgery is currently used to explore the events that are taking place in the retina for clinical research. However, several confounding factors such as vitreous haemorrhage and concentration of vitreous proteins should be considered in the analysis of the results. In this paper we will focus on the vitreous fluid as a tool for exploring the mediators of DR and in particular the molecules related to inflammatory pathways. In addition, their role in the pathogenesis of DR will be discussed. The usefulness of new technologies such as flow cytometry and proteomics in identifying new candidates involved in the inflammatory process that occurs in DR will be overviewed. Finally, a more personalized treatment based on vitreous fluid analysis aiming to reduce the burden associated with DR is suggested.

## 1. Introduction

Diabetic retinopathy (DR) remains the leading cause of blindness and vision loss among adults aged under 40 years in the developed world. Population-based studies suggest that about one-third of the diabetic population have signs of DR and approximately one-tenth have vision-threatening stages of retinopathy such as diabetic macular edema (DME) and proliferative diabetic retinopathy (PDR) [1–3]. DR is associated with considerable costs related to laser coagulation therapy, vitrectomy in severe cases, and eventually costs for social support when useful vision has deteriorated completely [4]. In this regard, it has been reported that the consumption of health care resources is almost double in type 2 diabetic patients with microvascular complications than in patients without it [5]. Notably, average healthcare costs increase considerably with the severity of DR, which suggests that preventing the progression of DR may alleviate the economic burden related to this complication of diabetes [6]. 

Current treatments for DR (laser photocoagulation, intravitreal corticosteroids, intravitreal anti-VEGF agents, and vitreo-retinal surgery) are applicable only at advanced stages of the disease and are associated with significant adverse effects [7–9]. Therefore, new pharmacological treatments for the early stages of the disease are needed.

The research in DR has three main limiting factors. First, a suitable animal model to explore both PDR and DME is needed. Among the available animal models, rodents have been studied most extensively owing to their short generation time and the inherited hyperglycemia and/or obesity that affect certain strains. In particular, mice have proven useful for studying DR and evaluating novel therapies because of their amenability to genetic manipulation. Mouse models suitable for replicating the early, nonproliferative stages of the retinopathy have been characterized, but no animal model has yet been found to demonstrate all of the vascular and neural complications that are associated with the advanced, proliferative stages of DR that occur in humans [10]. In addition, whereas most of clinical trials have been performed on patients with advanced DR, preclinical studies target prevention. Therefore, the success of a drug in preventing the development of experimental DR can hardly be transferred to the clinical practice. Second, the length of observation is another challenge. Although there is no fixed rule, the duration of the trial must be consistent with the natural history of DR and, in consequence, at least 5 years will be required to separate the behaviour of DR in the intervention and control groups. Finally, the direct access to the retina is not possible and for this reason vitreous fluid obtained from diabetic patients undergoing vitreoretinal surgery is currently used to indirectly explore the events that are taking place in the retina for clinical research. 

In this paper we will focus on the vitreous fluid as a tool for exploring the mediators of DR and in particular the molecules related to inflammatory pathways. 

## 2. Usefulness of Vitreous Fluid Analysis in Diabetic Retinopathy Research 

Regional concentrations of growth factors in the retina may be more important than systemic levels in the pathogenesis of DR. In this regard, vitreous fluid obtained from diabetic patients undergoing vitreoretinal surgery is currently used to indirectly explore the synthesis by the retina of mediators involved in the development of DR. Nondiabetic patients in whom vitrectomy is also indicated by conditions in which retina is not directly affected by neovascularization such as macular holes or idiopathic epiretinal membranes could serve as control group. However, there are two main confounding factors that could lead to misinterpretation of the results.

First, vitreous haemorrhage, which often occurs in PDR, can produce a massive influx of serum proteins, thus precluding the usefulness of the vitreous fluid when studying the intraocular production of a particular protein. This problem can be solved by either rejecting the vitreous samples in which haemoglobin is >5 mg/mL (measured by spectrophotometry) or adjusting the results using the equation proposed by Ambati et al. [11]. Second, the disruption of the blood-retina barrier (BRB) that occurs in DR produces an increase in proteins in the vitreous body of diabetic patients. Indeed, we have repeatedly detected 3-4-fold higher level of intravitreal proteins in diabetic patients than Nondiabetic subjects. Therefore, an elevated intravitreal level of a particular protein does not necessarily increase in intraocular production and might simply reflect a nonspecific increase in protein levels due to serum diffusion. This problem can be solved by either correcting the intravitreal concentration of the peptide under study for total vitreal proteins or calculating the ratio of vitreous to plasma concentration. This simple methodology has enabled us to rationalize the use of vitreous fluid as a tool for assessing the intraocular production of angiogenic, antiangiogenic factors and proinflammatory cytokines [12, 13].

Vitreous fluid obtained from PDR patients underwent vitrectomy only allows us to explore the mediators of advanced stages of DR. By contrast vitreous samples of cadaveric eyes obtained from diabetic patients without history of DR or who were free of fundoscopic abnormalities according to ophthalmologic examinations performed during the previous 2 years could be useful for exploring early stages of DR. We have used this strategy to demonstrate that downregulation of somatostatin is an early event of DR and is associated with retinal neurodegeneration [14]. Alternatively, vitreous samples obtained from diabetic patients without DR or with NPDR in whom vitrectomy is performed by a coexistent macular hole are also very useful. In fact, this approach permitted us to identify interphotoreceptor retinoid-binding protein (IRBP) as a new candidate in the development of DR [15].

## 3. Vitreous Inflammation in Diabetic Retinopathy 

Systemic inflammation is an intrinsic response to overfeeding, obesity, and diabetes, and diabetes increases the release of retinal inflammatory mediators and activation of microglial cells in early retinopathy [16].

A large body of evidence supports the role of proinflammatory cytokines, chemokines, and other inflammatory mediators in the pathogenesis of DR leading to persistent low-grade inflammation which contributes not only to the damage of the retinal vasculature but also to DME and PDR development [17, 18]. In fact, an emerging issue in DR research is the focus on the mechanistic link between activation of subclinical inflammation and angiogenesis [19]. 

### 3.1. Cytokines/Chemokines

Interleukin 1-*β* (IL-1*β*) is a pivotal inflammatory cytokine which is mainly produced by macrophage cells and it is able to activate NF-*κ*B [20]. Levels of IL-1*β* are known to be increased in retinas from diabetic rats. Intravitreal injection of IL-1*β*or exposure of retinal endothelial cells to the cytokine in vitro was shown to be capable of causing degeneration of retinal capillary endothelial cells [21]. In addition, IL-1*β* together with high concentrations of glucose (25 mM) has been used to induce the disruption of retinal pigment epithelial cells (outer blood-retinal barrier), thus mimicking what occur in DME [22]. However, the clinical relevance of these findings is not clear because the levels of IL-1*β*used in these in vitro experiments were much higher than those reported in vivo. 

The role of IL-1*β* in the pathogenesis of DR has recently been more directly studied using diabetic mice in whom the enzyme responsible for IL-1*β*production was inhibited or in whom the IL-1*β*receptor was deleted. IL-1*β*is the predominant product of caspase-1, and the biological activity of IL-1*β*is mediated by binding to the cell surface receptor, IL-1R1. Recent experimental evidence suggests that activation of caspase-1 and the subsequent production of IL-1*β*play an important role in the development of diabetes-induced retinal pathology [23]. 

Although IL-1*β*is essential in the inflammatory process involved in DR, there are few studies in which it has been found higher in the vitreous fluid of diabetic patients in comparison with Nondiabetic subjects [24, 25]. This is because the short half-life of the molecule and the low sensitivity of the commercial kits currently available. However, it should be noted that, as occurs with other cytokines, much of the IL-*β* production occurs at tissue level, where this production exerts important paracrine effects.

There are other interleukins that have been involved in the development of DR. Both interleukin-6 (IL-6) and interleukin-8 (IL-8) have been found elevated in the vitreous of patients with PDR [19, 24–29]. The role of IL-6 and IL-8 in the pathogenesis of PDR is not completely understood. However, there are reports suggesting that cytokine IL-6 can increase endothelial cell permeability in vitro by rearranging actin filaments and by changing the shape of endothelial cells [29]. IL-8 has been recognized as a potent chemoattractant and activator of neutrophils an T lymphocytes [30], and it is also a potent angiogenic factor [31]. In addition, it should be noted the mean levels for IL-8 within the vitreous fluid have been found in the same range as that reported in pleural effusions of patients with pneumonia or tuberculosis and they correlated with PDR activity [27]. Furthermore, the increased vitreous levels IL-6 and IL-8 correlated with the progression of PDR in the outcome of vitreous surgery [29]. These findings underscore inflammation as crucial in the pathogenic events that lead to PDR.

The source of high levels of IL-6 and IL-8 detected within the vitreous fluid of diabetic patients with PDR remains controversial. Plasma diffusion favoured by the breakdown of the BRB is an unlikely candidate. This is because of the strikingly higher concentrations of both cytokines detected in the vitreous fluid in comparison with serum. In addition, a relationship between plasma and vitreous concentrations of IL6 and IL-8 does not exist [27]. Thus, a possibility is that cells in the vitreous could be the main cause accounting for the high levels of these cytokines. In fact, macrophages, monocytes, retinal pigment epithelial (RPE) cells, and glial cells are found in the vitreous of patients with PDR, and the majority of these cells are capable of producing cytokines in vitro [32]. 

On the other hand, it is known that during the inflammatory reaction, anti-inflammatory cytokines are also produced and tend to modulate the inflammatory process. However, little information is available regarding the potential role of anti-inflammatory cytokines in PDR. Interleukin-10 (IL-10) is an anti-inflammatory cytokine with potent deactivating properties on macrophages. In addition, antitumoral effects of IL-10 have been associated with its ability to prevent angiogenesis by downregulating vascular endothelial growth factor (VEGF) expression. Our group provided evidence that this anti-inflammatory cytokine is not increased in the vitreous fluid of diabetic patients with PDR or, in other words, the enhancement of the proinflammatory cytokines is not counter-balanced by an increase of IL-10 [27]. 

### 3.2. Monocyte Chemotactic Protein-1

Monocyte chemotactic protein-1 (MCP-1) is the most common chemokine and its expression is regulated through NF-*κ*B. MCP-1 has been found elevated in the vitreous fluid of diabetic patients and their levels are higher than in serum [19, 25, 27–29, 33]. As occurs with IL-8, MCP-1 levels has been found in the same range as that reported in pleural effusions of patients with pneumonia or tuberculosis and they correlated with PDR activity [27]. Therefore, MCP-1 is a significant component of the diabetes-induced inflammation in the retina [34]. In fact, MCP-1 plays an important role in inducing leukocyte recruitment, and it is also a potent inducer of angiogenesis and fibrosis [35, 36]. Hyperglycemia has been shown to increase the MCP-1 generation from retinal vascular endothelial cells, RPE cells, and Muller's glial cells [28, 34]. Therefore, cells within the vitreous fluid could be the main cause accounting for the high levels of MCP-1 [32]. In addition, MCP-1 is expressed in myofibroblasts and in the vascular endothelial cells of epiretinal membranes from PDR patients [33]. Finally, the MCP-1 gene polymorphism has been indicated as a potential risk factor for DR, and the BRB disruption is prevented in a diabetic mice knockout for MCP-1 gene [37]. Further research is needed to establish the relevance of inhibitors of MCP-1 for preventing DR. 

### 3.3. Interferon Gamma-Induced Protein 10 (IP-10)

IP-10 is a CXC chemokine which has been found higher in the vitreous form diabetic patients than in Nondiabetic controls [33, 38, 39], and its levels have been reported even higher than those detected in serum samples [27]. The consequence of these findings is not easy to interpret because recent evidence demonstrates that members of the CXC chemokine family can act as either angiogenic or angiostatic factors, depending on the presence of the ELR (Glu-Leu-Arg) motif in their NH2 terminus [40]. Among this family, the chemokines IP-9/ITAC (CXCL11), MIG (CXCL9), CXCL4 (PF4), and IP-10 (CXCL10) lack the canonical N-terminal ELR sequence and bind in common to the ubiquitous CXCR3 chemokine receptor [41]. CXCR3 has two isoforms: CXCR3-A and CXCR3-B. Recent studies have shown that CXCR3 isoforms differentially regulate cell function. Activation of CXCR3-A has been shown to induce chemotaxis and proliferation in various cells types [42, 43]. Alternatively, CXCR3-B activation inhibits migration and proliferation and induces apoptosis [42, 44, 45]. There is emerging evidence showing that IP-10 mainly acts as an antiangiogenic factor via its signaling through CXCR3 [46, 47]. In addition, IP-10 inhibits angiogenesis in vivo at least in part by antagonizing the functions of IL-8 [48, 49]. Finally, an IP-10-derived peptide has been recently reported as a novel antiangiogenic agent [50]. For all these reasons, the elevated IP-10 levels detected in the vitreous fluid of diabetic patients could be contemplated as a mechanism to counteract the angiogenic effect of VEGF and other proinflammatory cytokines. 

### 3.4. Stromal Cell-Derived Factor-1 (SDF-1)

SDF-1 is the predominant chemokine which is upregulated in many damaged tissues as part of the response to injury and mobilizes stem/progenitor cells to promote repair [51]. SDF-1 acts through its receptor CXCR4 at several key steps in the process of ischemic repair, such as recruitment of endothelial progenitor cells (EPCs) from the bone marrow. Moreover SDF-1 induces VEGF expression in cells that are both hematopoietic and endothelial in origin, thus increasing the angiogenesis [52, 52].

SDF-1 works in conjunction with VEGF to promote the recruitment of endothelial progenitor cells (EPCs) from remote locations, such the bone marrow to the ischemic retina [53]. Butler et al. [52] demonstrated that SDF-1 concentration increases in the vitreous of patients with either DME or PDR, and this increase was correlated with disease severity. Notably, the levels detected within the vitreous fluid were able to induce DR in a murine model. Furthermore, the same group of investigators found a dramatic decrease in the intravitreous levels of both SDF-1 and VEGF after intravitreal injection of triamcinolone [52]. Taken together, these data demonstrate that SDR-1 plays a major role in the development of DR and may be an ideal target for future therapies. 

### 3.5. High-Mobility Group Box-1 Protein (HMGB1)

HMGB1 is a nonhistone DNA-binding that stabilizes nucleosome formation and facilitates transcription. Necrotic cell death can result in passive leakage of HMGB1 from the cell as the protein is then no longer bound to DNA. In addition, HMGB1 can be actively secreted by different cell types, including activated monocytes and macrophages, mature dendritic cells, natural killer cells, and endothelial cells. Recently, El-Asrar et al. [54] reported that HMGB1 and its receptor for advanced glycation products (RAGE) were expressed by vascular endothelial cells and stromal cells in PDR fibrovascular epiretinal membranes, and that there were significant correlations between the level of vascularization in PDR epiretinal membranes and the expression of HMGB1 and RAGE. They also demonstrated elevated levels of HMGB1 in the vitreous fluid from patients with PDR. 

Extracellular HMGB1 functions as a proinflammatory cytokine. When HMGB1 signals through RAGE, it leads to activation of NF-*κβ*, thus leading to the overexpression of proinflammatory molecules such as TNF-*α*, MCP-1, and ICAM-1 [54]. 

### 3.6. Tumor Necrosis Factor-*α* (TNF-*α*)

TNF-*α* is primarily synthesized by macrophages and T cells and its expression is regulated by NF-kB [55]. TNF-*α* is a cytokine that has been associated with the pathogenesis of several chronic inflammatory diseases including type 2 diabetes [56]. In fact, diabetic patients have higher TNF-*α* levels in serum than Nondiabetic patients, and a strong correlation between plasma levels of TNF-*α* and severity of DR has been reported [57]. However, as occurs with other cytokines, intraocular production of TNF-*α* could be more important than systemic levels in the pathogenesis of DR. In this regard, it should be noted that not only increased levels of TNF-*α* have been found in the vitreous fluid of diabetic patients [24, 26, 56, 57] but also a higher vitreous/serum ratio [26]. In addition, TNF-*α* has been found expressed in vascular endothelial cells and stromal cells in epiretinal membranes from PDR patients [19]. When analyzing TNF-*α* it should be considered its short half-life (~4 minutes), which could lead to false negative results. By contrast, soluble TNF-*α* receptors (sTNF-*α*-Rs) are more stable proteins, remaining elevated for longer periods of time and, therefore, being better markers of the activation of TNF-*α* system than TNF-*α* itself.

TNF-*α* is known to cause significant retinal endothelial permeability by PKC*ζ*-mediated downregulation of tight junction proteins and it is also required for VEGF-induced endothelial hyperpermeability, thus leading to the breakdown of the BRB which is the main pathogenic event of DME [58]. It also increases leukocyte adhesion and induces NADPH oxidase and production of reactive oxygen species (ROS) leading to retinal dysfunction of neurons and endothelial cells [39]. Finally, intravitreal injections of TNF-*α* into normal eyes lead to retinal ganglion cell death and optic nerve degeneration [59, 60]. 

For all these reasons, emerging strategies to block TNF-*α* actions in the diabetic eye seem warranted. Preliminary studies suggest a positive effect of intravenously administered TNF-*α* blockers [61, 62]. Unfortunately, much of the current data raises considerable safety concerns for intravitreal use of TNF-*α* inhibitors, in particular, intraocular inflammatory responses have been reported after intravitreal injection of infliximab. Results of dose-finding studies and humanized antibody or antibody fragments (e.g., adalimumab) are anticipated in the coming years; these will shed light on potential benefits and risks of local and systemic TNF-*α* blockers for treatment of DR.

### 3.7. Adhesion Molecules

There is growing evidence that leukostasis (the irreversible adhesion of leukocytes to the endothelium) plays a major role in capillary nonperfusion and retinal vascular leakage in DR [18, 63, 64]. In fact, intravitreal injection of corticosteroid attenuates the breakdown of the BRB by inhibiting leukostasis [65]. Moreover, leukocytes adhered to capillary endothelial cells induce apoptotic changes to endothelial cells [63, 64]. There is emerging evidence indicating that one of the most relevant mechanism by which leukocytes lead to the apoptosis of endothelial cells and the breakdown of the BRB is through the endothelial death via Fas-Fas ligand (FasL) [66]. In fact, suppression of Fas-FasL-induced endothelial cell apoptosis prevents diabetic BRB breakdown in a model of streptozotocin-induced diabetes. These data imply that the targeting of the Fas-FasL pathway may prove beneficial in the treatment of DR. 

Many of the cytokines detailed above lead to chemoattraction of inflammatory cells and consequently participates in leukostasis. Diabetic retinal vascular leakage, capillary nonperfusion, and endothelial cell damage are associated with leukocyte recruitment and adhesion to the retinal vasculature which correlates with increased expression of leukocyte adhesion molecules. 

The intercellular adhesion molecule ICAM-1 is the most important adhesion molecule in DR. The levels of ICAM-1 in the vitreous of patients with PDR are increased and the levels are higher in active PDR than inactive PDR [54]. Indeed, ICAM-1 is found to be highly expressed in the blood vessels of the retina, choroid, and fibrovascular membrane in patients with diabetes, and its expression correlates with the number of migrated neutrophils in the retina and choroid of these patients, thus indicating that elevated ICAM-1 facilitates leukocyte recruitment [67]. Furthermore, not only ICAM-1 levels are higher in diabetic patients, but also its ligands CD11a/CD18 and CD11b/CD18, specifically *β*-integrin and *α*-integrin [68]. Accordingly, the blockade of ICAM-1 or CD18 expression attenuates leukostasis, endothelial cell death, and vascular leakage in the retinal vessels of diabetic animals [69]. However, further investigation is still required to elucidate the role of integrin-ICAM-1 interaction in DR and the potential therapeutical benefits of its inhibition. 

Vascular cell adhesion molecule 1 (VCAM-1) and E-selectin are also involved in the pathogenesis of DR and their soluble forms has been found increased in the vitreous of PDR patients [70–72]. Both VCAM-1 and E-selectin can act on endothelial cells as angiogenic factors and a direct correlation between VCAM-1 and VEGF levels has been reported [71]. These findings suggest that therapeutic approaches aimed to block these soluble adhesion molecules could have beneficial effects on DR. 

Recently, the soluble vascular adhesion protein 1 (sVAP-1) has been found increased in the vitreous fluid and serum of patients with PDR [73]. It has been demonstrated that the retinal capillary endothelial cells produce the membrane-bound form of VAP-1 and release sVAP-1 when stimulated with high glucose or inflammatory cytokines such as TNF-*α* and IL-1*β*. The sVAP-1 seems to be involved in the pathogenesis of DR for two reasons. First, local expression of VAP-1 is involved in leukostasis and leukocyte entrapment [74]. Second, sVAP-1 has also an enzymatic function as a semicarbazide-sensitive amine oxidase which lead to the production of metabolites involved in cellular oxidative stress and advanced glycation end-product formation [73], two crucial events in the pathogenesis of DR. 

**Figure 1:**
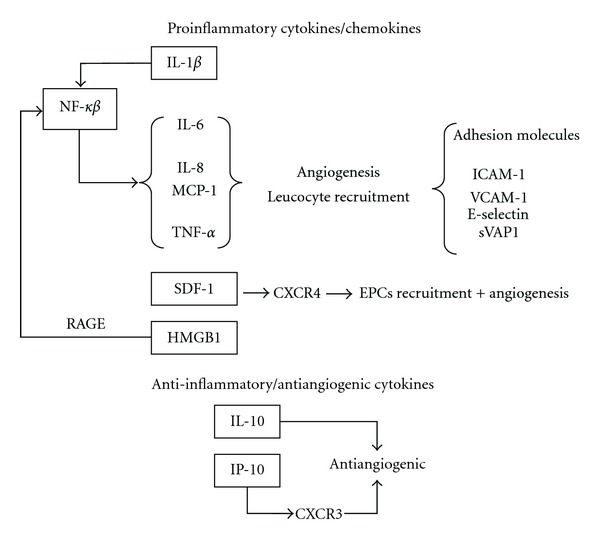
Main proinflammatory cytokines/chemokines increased in the vitreous fluid of diabetic patients (see text for details). Most of them participate also in the angiogenic process, which is essential for developing PDR. Anti-inflammatory/antiangiogenic cytokines also exist in the vitreous fluid of diabetic patients, but their concentration is not sufficient to counterbalance the inflammatory/angiogenic effect of proinflammatory cytokines.

In summary, an increase of several proinflammatory cytokines, chemokines, and adhesion molecules exists within the vitreous of diabetic patients which is not sufficiently counter-balanced by anti-inflammatory cytokines. This low-grade inflammation favours the angiogenic process. The main relationships among cytokines above mentioned are summarized in Figure 1. 

## 4. New Research Approaches 

### 4.1. Flow Cytometry

In Nondiabetic patients, the BRB has been shown to be impermeable to leucocytes. However, in the diabetic eye the migration of leucocytes into the vitreous body is favored due to leukostasis. One of the mechanisms involved is the alteration of adherent and tight junction proteins in the endothelial cells (i.e., proteolytic degradation of VE-cadherin) [75]. 

One of the major problems in any technique for studying the cells within the vitreous fluid is to obtain an adequate number for analysis. Many cells in the vitreous fluid are already nonviable, and the remainder can disintegrate very quickly after collection of the sample. Flow cytometry is a laser-based method of immunocytochemistry which permits a rapid and precise cell counting and sorting. Other benefits include easier cell preparation and multiparameter analyses of specimens. Earlier shortcomings including blood contamination, errors introduced by nonviable cells, difficulty in identifying monoclonality, and slow, single-cell suspension analysis, have been overcome. The main limiting factor is that the samples should be processed immediately. However, this allows us to simulate the in vivo scenario as close as possible.

By using this method, we found T lymphocytes in most of vitreous samples from PDR patients, whereas T lymphocytes were not present in the vitreous from Nondiabetic subjects [76]. This finding supports the concept that the disruption of the BRB is crucial for permitting the access of inflammatory cells into the vitreous body of diabetic patients. In addition, T cells infiltrating the vitreous shown a different pattern than in the peripheral blood (high percentage of CD4+ CD28−). Furthermore, those patients in whom T cells were detectable showed quiescent DR and their outcome was better than in those patients in whom intravitreous T cells were undetectable [76]. Therefore, it seems that T cells infiltrating the vitreous cavity have a protective role in the outcome of PDR. In this regard, it should be emphasized that the neuroprotective effect of autoimmune cells has been reported [77, 78]. In addition to anti-inflammatory cytokines like IL-10 or transforming growth factor, neurotrophic factors could be potential candidates to explain the protective effect of T cells on PDR outcome [79, 80]. 

Lipopolysaccharide-binding protein and soluble CD14 (sCD14) have been also found elevated in the vitreous fluid of patients with PDR and thus may play a role in the innate immune response triggered by the inflammatory injury characteristic of PDR [81].

The different pattern of T cells identified in the vitreous fluid of diabetic patients with PDR requires further functional characterization. In addition, further studies addressed to unraveling the intraocular innate immune defences that operate in PDR are needed. This research should provide a better understanding of the events involved in the development of immune response in DR and would help us in searching for more effective treatment for this disease.

### 4.2. Proteomics

The volume of vitreous fluid obtained after vitrectomy is approximately 1 mL and, therefore, only a few peptides can be analysed simultaneously. The recent development of proteome analysis has made it feasible to analyse protein profiles with only a small sample.

In recent years, several proteome analyses in human vitreous fluid have been reported in the setting of DR, thus permitting us to identify new potential candidates in its pathogenesis [82–89]. Regarding mediators of inflammation, it is worthy of mentioning that several factors of the complement system have been found increased in the vitreous fluid from PDR patients in comparison with control subjects [86, 88]. Activation of the complement cascade can both compound and initiate thrombosis, leukostasis, and apoptosis, all processes involved in vascular lesions of DR. Therefore, since several ways of specifically manipulating the complement system already exist, they could represent a possible therapeutic approach. Apart from complement factors, inflammation-associate proteins such as AAT, APOA4, ALB, and TF have been found significantly elevated in the vitreous of PDR patients [89].

**Figure 2:**
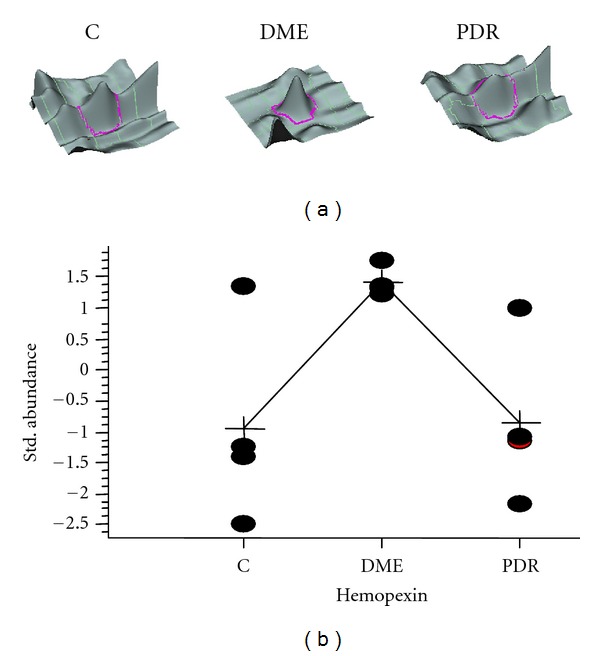
Results obtained by using the fluorescence-based difference gel electrophoresis (DIGE) strategy showing the higher abundance of hemopexin in the vitreous fluid of diabetic patients with DME in comparison with Nondiabetic controls and PDR patients. (a) Three-dimensional images of the hemopexin spot corresponding to the image of a control (C), DME, and PDR samples. (b) Standardised abundance plot for hemopexin displaying the log of abundance observed for the spot in each of the four gel images corresponding to control (C), DME, and PDR samples. The line links the average abundance values for each group of samples (crosses). Student's *t* test results in a significant increase (*P* < 0.05) in DME sample in comparison with either C or PDR sample.

Most of proteomic studies have been focused on PDR whereas there are only few studies performed on samples from patients with DME [90–92]. One of the most important findings of proteomic studies on DME has been reported by Gao et al. [91] demonstrating the essential role of both extracellular carbonic anhydrase-I and the kallikrein-mediated innate inflammation in the pathogenesis of DME. In addition, we have shown four proteins differently expressed in the vitreous fluid of patients with DME in comparison with PDR and Nondiabetic subjects: hemopexin (increased); clusterin, transthyretin, and beta crystalline S (decreased) [92]. Perhaps the most interesting finding is the increase of hemopexin (Figure 2). Hemopexin is an acute phase reactant which is believed to act as a protective molecule against heme-mediated oxidative injury as well as nitric oxide-mediated toxicity. Plasma hemopexin is mainly synthesized by hepatocytes, but it is also expressed by most of the cells of neural retina including the photoreceptors and, notably, the ganglion cells [93]. Apart from the elevated levels in the vitreous fluid on diabetic patients with DME, we have recently shown that hemopexin leads to the disruption of RPE cells, thus increasing permeability, and this effect is blocked by specific antihemopexin antibodies (unpublished results). Therefore, hemopexin could be a relevant factor in the pathogenesis of DME. T-cell-associated cytokines, like TNF-*α*, are able to enhance hemopexin production in mesangial cells in vitro, and this effect is prevented by corticosteroids [94]. Taken together, these findings suggest that hemopexin might be a mediator of the disruption of the BRB induced by proinflammatory cytokines, but further research on this issue is needed. 

## 5. Concluding Remarks and Future Research

Vitreous fluid is a useful tool for analyzing the pathophysiological events that are taking place in the retina of diabetic patients. However, several confounding factors such as vitreous haemorrhage and concentration of total vitreous proteins should be considered before validating the results. In addition, subjects who had undergone laser photocoagulation in the preceding 3–6 months should be excluded because a significant alteration in the balance of intravitreal growth factors and transcriptional activity in the retina has been shown following this procedure [95]. With all these caveats in mind, the analysis of key molecules involved in the pathogenesis of DR by using the vitreous fluid remains the most direct manner to explore the “in vivo” candidates involved in the development of DR. In fact, vitreous fluid analysis has been very useful in the translational research of DR. For instance, the seminal paper by Aiello et al. [96] in which was clearly demonstrated that VEGF was elevated in the vitreous fluid of PDR and it was able to stimulate retinal endothelial cells in vitro, as did vitreous fluid containing measurable VEGF, was essential for proposing anti-VEGF therapy by intravitreal injections in advanced stages of DME or PDR. Another more recent example is the low intravitreous levels of somatostatin detected not only in advanced but also in early stages of DR [14, 97–99]. These findings together with mechanistic experiments supporting the antiangiogenic and neuroprotective role of somatostatin have led to propose somatostatin as a replacement treatment for DR [100]. In this regard, a multicentric, phase II-III, randomized controlled clinical trial (EUROCONDOR-278040) to assess the efficacy of SST administered topically to prevent or arrest DR has been approved by the European Commission in the setting of the FP7-HEALTH.2011. This trial will start in September 2012 and the results should be available in 2015. 

Proinflammatory cytokines (i.e., IL-1*β*, IL-6, IL-8, TNF-*α*, and IP-10), chemokines (i.e., MCP-1, IL-8, IP-10, and SDF-1) and adhesion molecules (i.e., VCAM, ICAM, and VAP-1) have been found elevated in the vitreous fluid of diabetic patients, and the causal relationship between inflammation and angiogenesis is now widely accepted. Therapeutic strategies addressed to blocking their deleterious activity have been successfully reported in experimental models. However, the current treatment of both PDR and DME by intravitreous injections of anti-VEGF drugs or corticosteroids is not based in an individualized analysis. This is a serious limiting factor because the participation of either angiogenic factors (i.e., VEGF) or proinflammatory cytokines is highly variable in both PDR and DME. Therefore, a more personalized treatment based in the results of vitreous fluid analysis could be proposed.

New technologies such as flow cytometry and proteomics of the vitreous fluid have permitted us to gain new insights into the pathogenesis of both PDR and DME. Multiplex bead immunoassay, a type of assay that simultaneously measures multiple analytes in a single run/cycle of the assay, is also a useful tool in exploring the mediators of DR because it permits us to make the vitreous samples more profitable. Metabolomics has also allowed the obtainment a metabolic signature of PDR [101] and has the advantage of being applicable “in vivo” in the eye. With all these tools a more targeted treatment could be envisaged in the near future, thus reducing the burden associated with this devastating complication of diabetes.
